# Work stressors and their controllability: Content analysis of employee perceptions of hindrances to the flow of work in the health care sector

**DOI:** 10.1007/s12144-023-04328-3

**Published:** 2023-02-09

**Authors:** Heidi Lahti, Virpi Kalakoski

**Affiliations:** 1 Finnish Institute of Occupational Health, Helsinki, Finland; 2 Faculty of Social Sciences (Psychology), Tampere University, Tampere, Finland

**Keywords:** Hindrance stressors, Flow of work, Stressor controllability, Work stress, Health care sector, Content analysis

## Abstract

High levels of work stress are prevalent today, and the underlying working conditions need to be tackled urgently. In this study, our aim was to identify the range of factors that employees themselves perceive as hindrances to the flow of work, that is, hindrance stressors. We analysed the open-ended questionnaire responses of 4766 employees working in the health care sector using semi-automated content analysis. We then used more detailed conventional content analysis to compare the responses of the groups that reported high (n = 1388) and low (n = 833) levels of subjective stress. Finally, we interpreted and categorised the stressors raised by the respondents from the viewpoint of controllability, to shed light on where to target interventions. The main hindrance stressors reflected inadequate staffing, work overload, time pressure, and management-related issues, of which the responses revealed concrete examples. Interruptions and problems related to cooperation and instructions were also commonly mentioned. The respondents in the high stress group emphasised work overload and issues related to management and clients. Our results suggest that the major hindrances to daily work are beyond employees’ control and require decisions and resources at the level of supervisors, managers, directors, and policymakers. Future studies on work stress should explore the controllability of common stressors in more detail and include the appraisal of controllability in explanatory models. Avoiding overemphasis of psychological coping and instead targeting harmful working conditions and the organisational actors who can influence these could make workplace stress management interventions more effective.

## Introduction

High levels of stress are prevalent in many occupations (Johnson et al., 2005), and recent studies suggest that these levels have increased in many western countries in the last few decades (e.g., Almeida et al., 2020; Rigó et al., 2021). Prolonged stress is associated with many adverse health outcomes, such as burnout, depression, and cardiovascular diseases (Fishta & Backé, 2015; Kivimäki et al., 2002; Maslach et al., 2001; Tennant, 2001), and it also imposes a major economic burden on society and organisations in the form of health care costs and lost productivity (Béjean & Sultan-Taïeb, 2005). Given the high prevalence of chronic stress and its wide-ranging adverse consequences, it is not surprising that the causes and management of work stress have attracted growing interest among researchers and occupational health practitioners in recent decades. Nevertheless, despite the extensive literature on the causes of chronic stress, there is little information about what the day-to-day stressors at workplaces are that most urgently should be managed to reduce the adverse outcomes of stress (Lukan et al. 2022).

In the currently most influential models of work stress, high job demands are understood as work environment risk factors that may cause chronic stress on employees. The Job Demands-Resources (JD-R) model states that job strain develops when high job demands are accompanied by limited job resources, such as low job control and social support (Bakker & Demerouti, 2007; Karasek, 1979). Recent conceptualisations based on the Challenge-Hindrance Stress Model (CHM) originally introduced by Cavanaugh et al. (2000) have further categorised job demands along with situational constraints at work as either challenge or hindrance stressors (Bakker & Demerouti, 2017; Mazzola & Disselhorst, 2019, Schaufeli & Taris, 2014). Whereas challenge stressors are job demands that are appraised positively by employees as rewarding and motivating, hindrance stressors are defined as job demands or working conditions that involve excessive or undesirable constraints which in turn interfere with or hinder employees’ abilities to achieve their goals (Cavanaugh et al., 2000).

Recent conceptualisations thus indicate, and empirical findings support, that job demands appraised as negative hindrances are risk factors for work stress (Mazzola & Disselhorst, 2019). Nevertheless, most earlier studies have a major pitfall: they have used a priori classifications of stressors as challenges or hindrances (Horan et al., 2020). Many scholars have criticised this practice, arguing that the same job demands may be appraised either negatively or positively in different work contexts and by different individuals (Bakker & Demerouti, 2017; Mazzola & Disselhorst, 2019, Schaufeli & Taris, 2014). For example, in certain occupations, work pressure is perceived as a hindrance stressor even though workload and time pressure are often classified a priori as positively experienced challenge stressors (Bakker & Sanz-Vergel, 2013). Furthermore, a recent study by the authors demonstrated that although most employees appraised time pressure, for example, as a strenuous hindrance, large groups of employees appraised many cognitive job demands that are frequent in daily work, such as demands for attention or learning, as both energising challenges and strenuous hindrances (Kalakoski et al., 2022). Thus no universal appraisal exists. To sum up, current research does not provide a comprehensive understanding of which of the many job demands and concrete situations in daily work may be perceived as hindrance stressors and thus require stress management interventions.

Despite the vast research literature on work stress, previous research offers limited insight into how the factors identified as harmful stressors manifest in daily work life and cause “day-to-day stress”, that is, how various situations at work translate to an experience of stress (Lukan et al., 2022). Moreover, as current models of job demands focus on specific sub-dimensions and measures of job demands, they may overlook other relevant risk factors (Burr et al., 2016). Therefore, the current study will explore in more detail, using extensive qualitative data, the factors that employees themselves, as experts of their own work, perceive as the main hindrance stressors. These are the issues that most acutely call for interventions and pose a threat not only to employees’ wellbeing but also to the smooth flow of work.

Thus far, most workplace stress prevention efforts have aimed to improve individual-level resources such as employees’ stress management skills or health behaviours (Grawitch et al., 2015; Nikunlaakso et al., 2022; Semmer, 2006). In this study, however, we advocate stress prevention that focuses on creating working conditions that do not induce undue amounts of stress (Fox et al., 2021; Semmer, 2006) and aim to identify the hindrance stressors that underlie the experience of stress. Indeed, reducing job demands that employees experience as hindrance stressors is potentially a highly effective approach for stress prevention (e.g., Rickard et al., 2012) and has often been recommended in recent literature (e.g., Shoman et al., 2021). Therefore, it is essential to listen to employees with regard to the stressors that they perceive as common sources of unnecessary strain, as these constitute the issues that interventions should target.

Finally, if the aim is to change the actual working conditions that underlie stress, it is of utmost importance that we identify who controls these issues, so that we can target interventions at the right actors. Modern (neuro) physiological stress research has acknowledged that the uncontrollability of stressors is a major contributor to the onset of stress mechanisms that lead to harmful physiological and behavioural responses (Koolhaas et al., 2011; Limbachia et al., 2021; Meine et al., 2021). Psychological models of work stress have also long recognised autonomy (and the related job characteristics termed job control or decision latitude, depending on the model) as one of the most important resources that may buffer the effects of job demands on strain (e.g., Bakker & Demerouti, 2007; Karasek, 1979). However, the kind of autonomy discussed in the work stress literature usually concerns autonomy over work methods, work scheduling, or time and place of work (e.g., De Spiegelaere et al., 2016), and not the factors often recognised as major stressors, such as the amount of work (Colligan & Higgins, 2006). Therefore, it is unclear whether and to what extent the main causes of workplace stress are under the control of employees. The current study helps us understand which main hindrance stressors employees face in their daily work, and whether the employees themselves or other organisational actors can control these stressors. The results will yield new insights into psychological work stress research that aims to develop effective interventions to manage the hindrance stressors in daily work.

### Context and aims of present study

As highlighted above, we need research that explores, without any pre-defined response options or categories, the various factors that are appraised as hindrance stressors by employees in different occupational settings. The current study focuses on the health care sector, in which employees report above-average levels of subjective stress (e.g., Cooper, 1999; Young & Cooper, 1999) and are at a heightened risk of burnout (Cañadas-De la Fuente, 2015). We asked a large, diverse group of employees working in the Finnish health care sector to provide a free-text response to a question on the factors they perceive as hindrances to the flow of work, that is, as hindrance stressors. With data consisting of thousands of responses, we were able to adopt an exploratory data-driven approach and apply a semi-automated content analysis that uses quantitative information on word occurrence and co-occurrence to comprehensively map the variety of hindrance stressors as perceived by the employees.

To obtain an even more detailed picture of these factors and their relative significance from the viewpoint of employee wellbeing, we further supplemented the analysis of qualitative data with an element of quantitative comparison, an approach neither extensively nor sufficiently utilised in previous studies (Lindsay, 2019). We used a validated measure of self-reported stress to divide the respondents into high and low stress groups. We then used conventional content analysis to study whether the employees who reported high levels of stress emphasised any specific hindrance stressors in comparison to employees who reported low stress. Finally, we examined the categories from the viewpoint of controllability, that is, we sought to determine the organisational levels and actors who have control over the stressors mentioned in the responses. Most previous research has conceptualised job control as a broader-level job resource. In this study, however, we took a novel, more concrete approach and focused on controllability as a characteristic of day-to-day work stressors.

The study illuminates the nature of common hindrance stressors from the perspective of employees’ daily work and has practical value for future workplace stress management interventions. Our overall aim was to gain new insights into the hindrance stressors that most loudly call for interventions at work, and to identify the actors who can influence working conditions that may lead to harmful work stress. To our knowledge, this is the first study to address how thousands of employees express the factors they perceive as hindrance stressors in open-ended responses. Furthermore, our mixed-method research design enabled us to compare the responses of large groups of employees with high and low levels of subjective stress. This design responds to recent calls for greater use of qualitative and mixed methodology approaches in occupational stress research, to provide a deeper understanding of the stress process and of employees’ personal experiences (Horan et al., 2020; Mazzola et al., 2011).

Our research questions were:

1. What are the major hindrance stressors perceived by employees working in the health care sector?
2. Are there differences between the relative frequencies of the various hindrance stressors described in the responses of employees reporting high levels and those reporting low levels of subjective stress?
3. On what level of the organisation can the hindrance stressors mentioned in the responses be controlled (individual employee, work team, supervisor, manager, director, policymaker)?

Answering these questions will help future stress management interventions and workplace development focus on the issues that most need improving and on the actors who can influence these conditions.

## Materials and methods

This study was part of the *Five approaches to brain work* project (Viisikko) conducted at the Finnish Institute of Occupational Health (FIOH). It was based on extensive, anonymous FIOH Brain Work data gathered through surveys during 2016–2019 in the context of various research, development, and service projects. The data consisted of responses form more than 11,000 employees from different industries and from more than 90 separate surveys.

The original surveys had various sets of items, relating to background information (such as age, gender, and field of work), the nature of cognitive work (the FIOH Brain Work Questionnaire, BWQ), and items relating to working conditions and well-being. In this study, we used a subset of the larger data set, which included survey responses collected in organisations operating in the Finnish health care sector (14 separate surveys). In the analyses two items from the survey were used: an open-ended question on the hindrances to the flow of work and a quantitative question on subjective stress.

### Sample

The subset of the data used in this study consisted of the responses of 5982 employees. Of these respondents, 4766 (79.7%) had replied to the open-ended question that was the focus of this study, and this comprised the sample we used in the semi-automated content analysis. The sample used in the conventional content analysis consisted of respondents who reported relatively high (n = 1388) or low (n = 833) levels of stress (details on how the groups were defined are provided below, under the heading “Survey items”). These respondent subgroups comprised 29% and 17% of the total study sample, respectively.

At least 91% of the respondents provided background information on various aspects. The respondents were from all the 19 regions in Finland, and about two-thirds of them were employed by a public sector organisation (city, municipality, or federation of municipalities), and about one-third worked for either a private sector company or a foundation. There were no differences between the high and low stress groups in terms of geographical location or type of employer. The majority of the respondents worked either with the elderly in sheltered housing or home care (62%) or in hospitals or health centres (14%). The rest of the respondents came from a variety of specialisation fields, such as mental health and substance abuse services and occupational health services. The employees who worked with the elderly were slightly over-represented in the high stress group (66% vs 54%), and the employees working in hospitals or health centres were slightly over-represented in the low stress group (18% vs 12%).

Most respondents in both groups were women (96%) and their mean age was 47.9 (SD = 11.0) years. The respondents were slightly younger in the group that reported high stress (M = 46.8, SD = 11.5) than those in the group that reported low stress (M = 49.5, SD = 9.9).

The majority of the respondents had a (vocational) upper-secondary education (53%). Also, a large proportion had either a post-secondary education (33%) or an equivalent polytechnic education (6%). A minority had only attended comprehensive school (7%) or had a university or higher-level degree (2%). The proportion of respondents with a (vocational) upper secondary education was somewhat pronounced in the high versus the low stress group (54% vs 48%), whereas higher education level (post-secondary/polytechnic/university) was somewhat pronounced in the low versus the high stress group (45% vs 39%).

### Survey items

The survey included a single-item measure of stress (see Elo et al., 2003 for a validation study). The question was phrased “By stress we mean a situation in which a person feels tense, restless, nervous, or anxious, or they find it difficult to sleep because they cannot switch off their thoughts. Do you currently feel this kind of stress?”, and the respondents were asked to assess their situation on a scale of 0 to 10, where 0 meant not at all and 10 meant very much. The high and low levels of stress were defined on the basis of the distribution of the item values in the entire Brain Work data (n = 11,058). The highest quarter of responses included item values of 8, 9, or 10, and these were classified as high-level stress, whereas the lowest quarter of responses included item values of 0, 1, and 2, which were classified as low-level stress. The mean of the reported stress in the study sample (M = 5.62, SD = 2.78) was slightly higher than that in the larger Brain Work data (M = 5.34, SD = 2.74).

Another survey item used in this study was an open-ended question at the end of the survey which asked the respondents to write about the factors they perceived as hindrances to the flow of work and sources of unnecessary strain. The question was phrased in two slightly different ways. Most of the respondents (91%) responded to “What factors do you think hinder the flow of work and cause unnecessary strain?”, and the rest responded to “What do you think are the main hindrances to the smooth flow of work?”. The respondents wrote their responses in a digital survey box which did not limit the length of the response. All the items and responses were originally in Finnish.

### Qualitative data and analyses

**Fig. 1:**
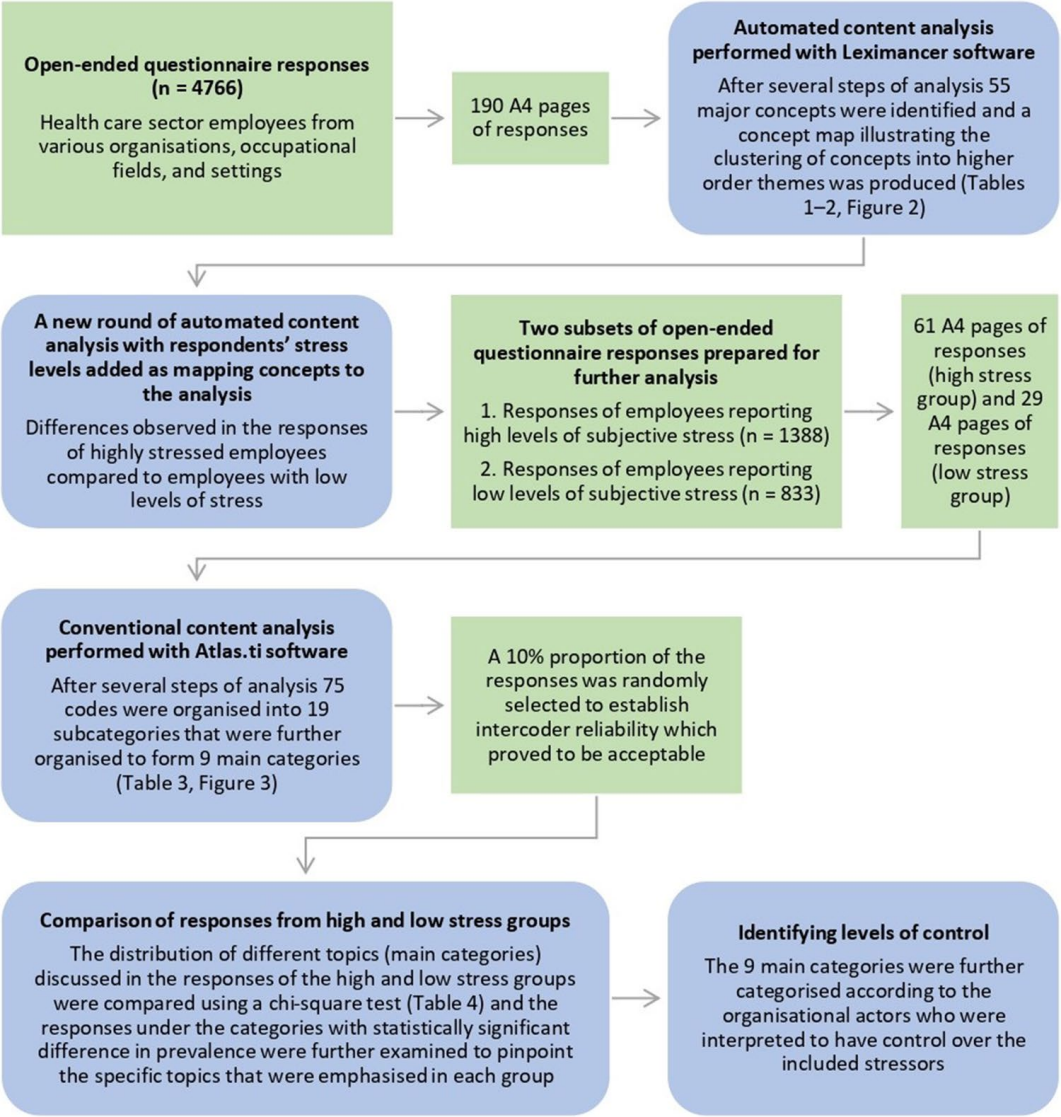
Analysis process

Figure 1 shows the overall data analysis process. The textual responses were formatted so that they could be analysed using Leximancer 4.5 and ATLAS.ti 9.0 software. There were altogether 60,109 words of text (190 tightly spaced pages) and the individual responses varied greatly from short one-word answers to more elaborated narratives. The total number of words produced in the high stress group was 20,522 (61 pages), and in the low stress group 8516 (29 pages).

We used two different approaches to analyse the responses. First, we used a computer-assisted quantitative approach based on word frequencies and lexical co-occurrence information. The term semi-automated content analysis is used to refer to this approach. Second, the first author conducted a manual segmenting and categorising of the text, and the term conventional content analysis is used to refer to this second more traditional qualitative approach.

We chose to combine these two content analysis approaches for two reasons. First, automated analysis largely avoids the researcher bias inherent in human-performed coding (Sotiriadou et al., 2014). It is known that achieving a high degree of reliability in traditional human-coded content analysis is not easy, especially when large amounts of textual data are processed, as this increases the likelihood of errors (Su et al., 2017). Second, complementing the semi-automated analysis with a more conventional method when comparing the high and low stress groups enabled more explicit categorisation of response content and provided a more detailed picture of the differences between the groups. For example, we obtained more detailed information on the number of responses that dealt with specific topics in each group. Some researchers have even recommended combining automated and manual text analysis (Sotiriadou et al., 2014), as the two approaches complement each other and together provide a more comprehensive picture of the data (Wilk et al., 2019).

We first analysed the entire set of responses using Leximancer, a text mining software that utilises machine learning to automatically identify the main concepts and their relations in textual data. Leximancer enables the analysis of large volumes of text and largely avoids the researcher bias inherent in human-performed coding, because the manual interaction required from the researcher is minimal (Cretchley et al., 2010). Leximancer analysis has shown to have satisfactory reliability, stability, and face validity (Smith & Humphreys, 2006). The software has been utilised extensively in various fields of research (e.g., Dambo et al., 2021), and several work and organisational research papers utilising Leximancer have also been published (e.g., Arasli et al., 2020; Fruhen et al., 2013).

In the Leximancer analysis, both semantic and relational co-occurrence information are extracted from the text by two separate statistical algorithms (Smith & Humphreys, 2006). Thus, information is provided on both the presence of the identified concepts (i.e., a group of words occurring together throughout the text) and on the interrelations of these concepts. The analysis was conducted exploratively using unsupervised analysis. The text segment size was set to two sentences and we used the default Finnish stop word list, with some additional stop words. We also fine-tuned the concept list, a necessary procedure in unsupervised content analysis by Leximancer (Crofts & Bisman, 2010). This fine-tuning meant that, for example, synonyms, plurals, and words with similar meanings were merged (e.g., staff and employees). The applied Leximancer analysis procedure is described in more detail in the Appendix, and a more detailed description of the logic underlying Leximancer can be found in Smith and Humphreys (2006).

Second, we conducted conventional content analysis (see Hsieh & Shannon, 2005) to examine the responses of the groups reporting high and low levels of stress (a smaller portion of the data). We followed the phases of inductive content analysis, an approach outlined in Elo and Kyngäs (2008) and recommended when the prior knowledge of a phenomenon is fragmented. In addition to its main purpose of enabling comparison of the stress groups, conventional content analysis also served the purpose of complementing and elaborating the results of the semi-automated Leximancer analysis.

In the conventional approach, the responses were manually coded using ATLAS.ti. The responses were segmented on the basis of the discussion topic, and the size of the resulting segments varied from single words to several sentences. In the coding process, the coding categories were derived directly from the text in an inductive manner so that new categories were continuously and iteratively created as new topics were encountered. The text segments were multiply coded into all the coding categories that they reflected. The first author coded all the responses (n = 2221) using an emerging coding scheme which, in the end, contained 75 individual codes. The proportion of unclassified text was less than 2%.

Ten per cent of the responses from both stress groups was randomly selected to sufficiently establish intercoder reliability (O’Connor & Joffe, 2020). We calculated simple percent agreement on the level of the nine main categories (described in detail in the Results section), as this was the level on which all the results involving numbers were reported. The percent agreement was 78%, a level that has been suggested to indicate acceptable reliability (O’Connor & Joffe, 2020). A more detailed description of the applied analysis procedure is available on request from the authors.

## Results

### Overall picture of the hindrance stressors

In the Leximancer analysis 55 concepts were identified. Table 1 presents the most central concepts (those with relevancies of at least 10%), their counts and representative quotations from respondents. The results revealed a variety of factors and conditions hindering the flow of work that were repeatedly mentioned in the responses.

**Table 1:** Most central concepts, counts, relevancies (i.e., concept’s count in relation to most frequently appearing concept), and representative quotes

Concept	Count	Relevance	Representative quotes
Employees	1556	100%	“Shortage of employees.”
Time pressure	1001	64%	“Time pressure, doing many things at the same time.”
Shortage	756	49%	“Shortage of workers in the shifts.”
Constant	657	42%	“Constant time pressure.”
Poor	603	39%	“Poor workplace atmosphere.”
Work tasks	557	36%	“Several work tasks pile up simultaneously.”
Substitutes	455	29%	“Constantly changing substitutes or the fear of not getting one.”
Lack	425	27%	“Lack of employees in almost every shift.”
Clients	404	26%	“Clients’ health has deteriorated.”
Instructions	398	26%	“Insufficient or complicated instructions.”
Much	394	25%	“Too much work and not enough time.”
Supervisor	342	22%	“Poor and unjust supervisor.”
Time	330	21%	“Not enough time for all tasks.”
Unclear	295	19%	“Unclear instructions.”
Changes	261	17%	“Constantly changing instructions and rules from the employer.”
Interruptions	257	17%	“Interruptions to work that requires intense concentration.”
New	246	16%	“New duties constantly assigned in addition to old ones.”
Do	241	15%	“Having to do many different things at the same time.”
Changing	200	13%	“Constantly changing co-workers.”
Information flow	178	11%	“Poor information flow.”
Noise	158	10%	“Noise and commotion, office work is constantly interrupted.”

The words frequently appearing in the text serve as “concept seed words” around which the Leximancer algorithm defines the concepts and the keywords associated with each one (for up to hundreds of words). For example, the thesaurus of the “clients” concept includes keywords such as “client”, “residents”, and “elderly”

**Fig. 2:**
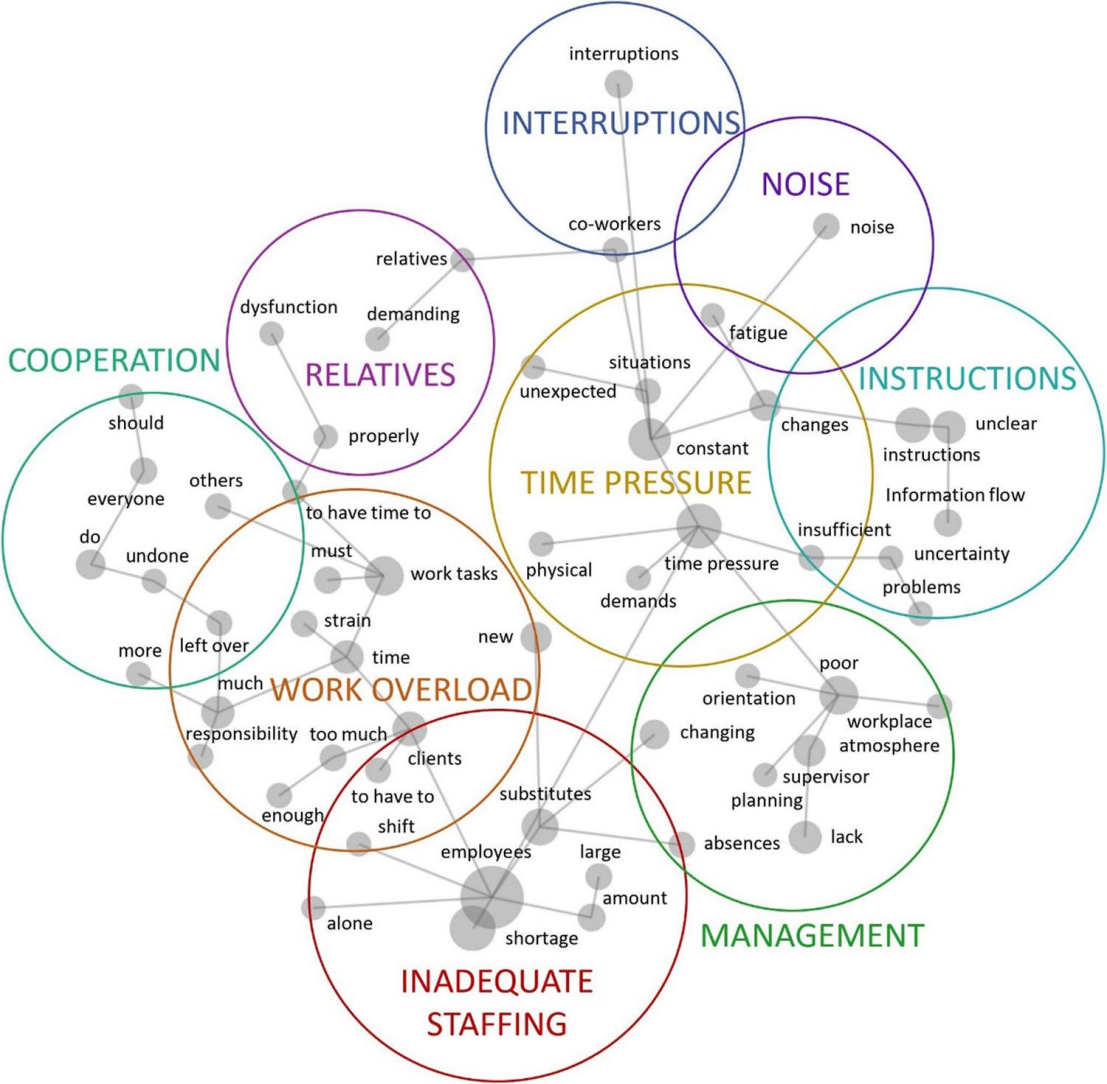
Leximancer concept map of major hindrance stressors as perceived by employees. Each of the 55 concepts is represented by a grey dot, the size of which signifies its connectivity to all the other concepts. The spanning tree of grey lines represents the strongest connections between the concepts. Concepts frequently appearing together in the text are clustered into themes, indicated by the large circles

The results of the Leximancer analysis are visualised as a concept map that illustrates all the 55 concepts identified in the responses in terms of prevalence and interconnectedness, as well as the higher level “themes” into which frequently co-occurring concepts were clustered (Fig. 2). The proximity of the concepts on the map indicates how often they appeared together in the text. For example, 88% of all the text segments that contained the concept of “shortage” also contained the concept of “employees” – thus these two concepts are located close to each other on the map. Similarly, 75% of all the text segments that contained the concept of “instructions”, also contained the concept of “unclear”.

The concept map in Fig. 2 also illustrates how the concepts were clustered to form nine themes. The themes were named after careful consideration of the occurrence and co-occurrence information of the concepts grouped into each one. Most themes were named after the most connected concept within its circle. The theme circles are heat-mapped such that the colour represents the importance of the theme. The most relevant themes are denoted by hot colours (red, orange) and the least relevant themes with cool colours (blue, purple). Table 2 provides the same information in a more detailed numerical form, presenting the number of hits for each theme (i.e., relevance, total number of text segments associated with that theme), and representative quotations from respondents.

**Table 2:** Themes and associated number of hits in Leximancer analysis, and representative quotes

Theme	Hits	Representative quotes
Inadequate staffing	2037	“New substitutes all the time and not enough time to teach them the tasks. Or working understaffed, often alone on a shift.”
		“Too little trained staff. Large number of care assistants.”
Work overload	1866	“If there are too many clients for one day, things need to be taken care of quickly. And as we are working with people, every nurse should have enough time for actual nursing.”
		“Lots of new employees as co-workers whom I have to teach and help, and this burdens me. I like working in a profession that has co-workers of different ages, but my own tasks and instructing a co-worker takes a lot of time.”
Time pressure	1773	“Constant time pressure and stress, physically strenuous work. Constant changes and unpredictable events at work.”
		“Time pressure, changing situations and a high number of absences.”
Management	1505	“Poor planning of tasks, lack of information about new things, poor management, poor workplace atmosphere.”
		“Supervisor’s unrealistic understanding of everyday work life and of how working hours can be used.”
Cooperation	678	“There are no established practices at work, everyone works in their own way. Some neglect their own tasks, [and] they are left to others.”
		“You never have time to finish even mandatory tasks during the workday. [Medical record] entry details are often left unfinished.”
Instructions	629	“Unclear instructions [and] unawareness of responsibilities [and] tasks. Interaction problems that we don’t know how to solve.”
		“Unclear instructions, when information is not shared in the organisation and there is no time to write down instructions, etc.”
Interruptions	364	“Constant interruptions.”
		“Enormous number of interruptions and many tasks in progress all the time, difficult to get anything finished in one go.”
Noise	206	“Noise, people shouting from one room to another”
		“Constant noise and restlessness”
Relatives	171	“Patients and their relatives have all kinds of demands and questions that interrupt and slow down work.”
		“If technology does not function properly.”

The most relevant and strongly interconnected themes discussed in the responses were *Inadequate staffing*, *Work overload* and *Time pressure,* and these formed the core of the entire set of responses. The smaller themes of *Cooperation* and *Relatives* were also located close to the theme of *Work overload*. The entwined nature of these topics is also evident in the respondent quotations listed in Table 2, which paint a picture of everyday work in which inadequate staffing levels, excessive workload, and time pressure, as well as excessive workload and difficulties in cooperation are intertwined.

On the top right of the concept map, the smaller themes of *Instructions*, *Interruptions* and *Noise* formed another, qualitatively different cluster around the central theme of *Time Pressure*. The related concepts and example quotations describe unpredictable and constantly changing situations at work, unclear instructions, as well as interruptions and noise that impair the flow of work.

Finally, the central theme of *Management* also widely connected to other themes; both the cluster comprising *Inadequate staffing* and *Work overload* and the cluster comprising *Time pressure* and *Instructions*. Problems concerning the planning of work, orientation, and workplace atmosphere were examples of concepts that clustered under the *Management* theme.

### Differences between high and low stress groups

The groups reporting high and low levels of stress differed in terms of the average length of the individual responses, which was 12.6 words in the total sample. The responses in the high stress group were on average longer (14.8 words) than those in the low stress group (10.2). Moreover, the employees in the high stress group tended to bring up more topics (i.e., average number of codes applied to an individual response) than those in the low stress group (2.7 vs 1.9 different topics, respectively).

The high and low stress groups were first compared by adding the stress group classifications as mapping concepts to the Leximancer analysis. The comparison revealed that all the 55 concepts were present in the responses of both stress groups, and the most prevalent concepts largely overlapped in the two groups. However, the analysis also showed that the high stress group was located on the concept map closer to the *Inadequate staffing*, *Work overload*, and *Management* themes, whereas the low stress group was located on the other side closer to the *Cooperation* and *Interruptions* themes. Differences between the high and low stress groups were also evident in the relative prevalence of the concepts. In the responses of the high stress group, for example the “supervisor”, “demands”, “demanding”, “constant”, “clients”, and “workplace atmosphere” concepts were relatively more frequent than in the low stress group. On the other hand, the “co-workers”, “others”, “absences”, “undone”, “interruptions”, and “noise” concepts were relatively more frequent in the responses of the low stress group than in those of the high stress group.

**Fig. 3:**
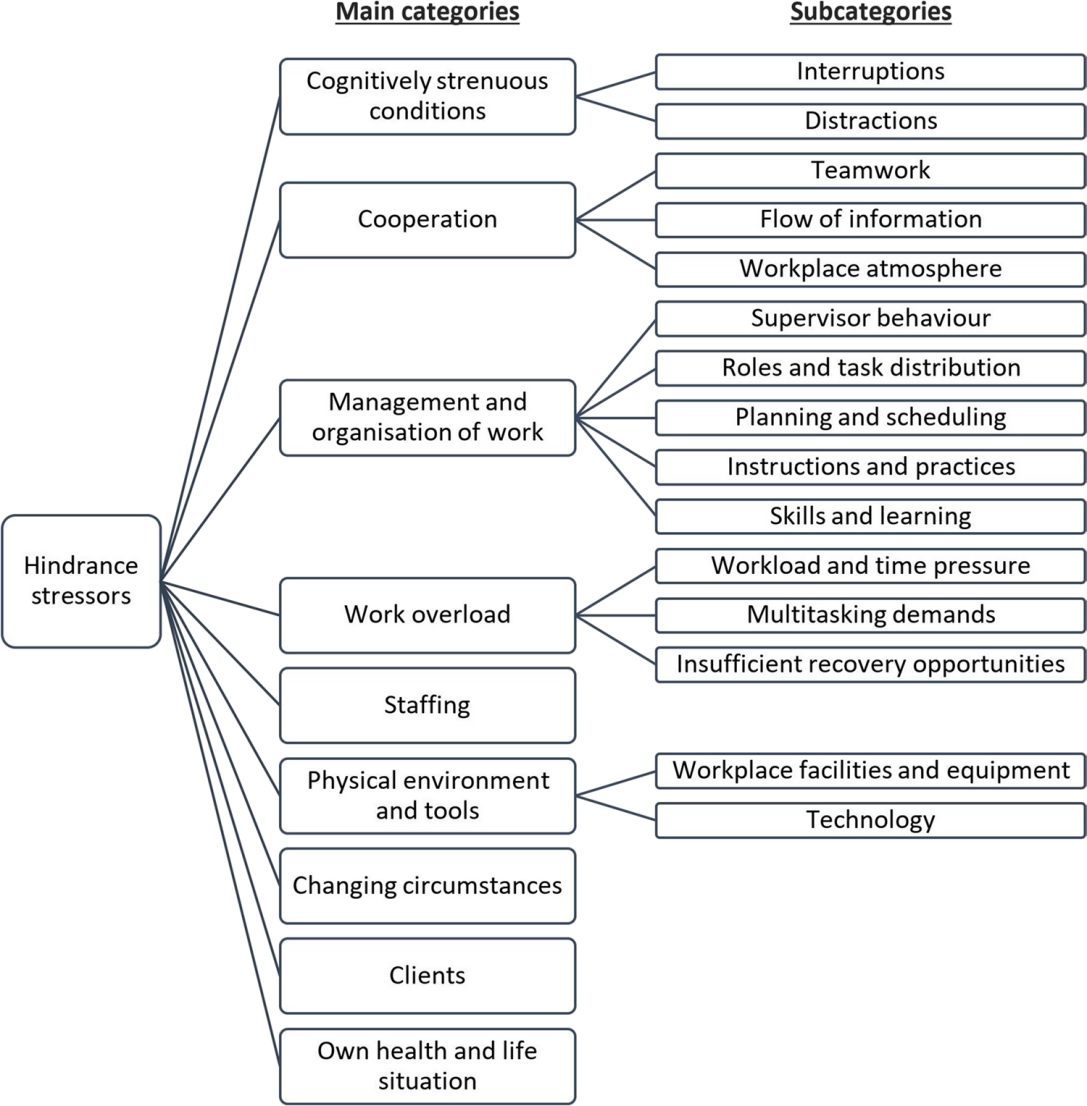
Classification of hindrance stressors into nine main categories and related subcategories

The differences between the high and low stress groups were studied in more detail using conventional content analysis. The 75 codes originally applied to the responses were organised into 19 categories, the descriptions of which are presented in Table 3. These initial categories were further grouped into nine broader-level main categories five of which included several subcategories (Fig. 3).

**Table 3:** Main categories, subcategories, and descriptions of their key content

Main category	
Subcategory	Description of key content
Cognitively strenuous conditions	
Interruptions	Constant interruptions of work by co-workers/clients/phone calls
Distractions	Noise, restlessness, having no space for quiet work
Cooperation	
Teamwork	Poor teamwork, co-workers’ lack of contribution/responsibility-taking
Flow of information	Poor communication/flow of information, insufficient medical record entries
Workplace atmosphere	Poor workplace atmosphere, inappropriate behaviour such as bullying, fatigued co-workers
Management and organisation of work	
Supervisor behaviour	Supervisor’s behaviour/attitudes/demands, lack of support from management
Roles and task distribution	Wide range of duties and responsibilities, uneven distribution of work, working alone
Planning and scheduling	Poor planning of employee shift schedules/work, not enough time reserved for tasks
Instructions and practices	Unclear/changing instructions, lack of common instructions/practices/work methods
Skills and learning	Deficient skills of co-workers, lack of job orientation/opportunities for training and learning
Work overload	
Workload and time pressure	Constant time pressure, too much work, unfinished work, working overtime
Multitasking demands	Having to perform multiple tasks at the same time, too many things to simultaneously remember
Insufficient recovery opportunities	Not having time to take breaks/eat lunch, not having enough time off work
Staffing	Inadequate staffing levels, employee shortages, employee turnover, substitute employees
Physical environment and tools	
Workplace facilities and equipment	Inadequate working facilities or work equipment, poor indoor air quality
Technology	Malfunction/deficiency/number of technologies used, new programmes/devices
Changing circumstances	Unpredictable and changing situations in everyday work, constant change in general, uncertainty
Clients	Poor health of clients, challenging or demanding clients and relatives
Own health and life situation	Employee’s own poor health, fatigue, difficult life situation

After applying the codes and reading through the material several times it was evident that basically the same spectrum of issues was brought up by employees with high and low stress levels, but the relative prevalence of different topics varied between the groups. The distribution of different topics (main categories) discussed in the responses of the high and low stress groups were compared using a chi-square test to analyse whether there were differences between the two groups. The results showed that the distribution of the nine main categories differed in the two stress groups, χ^2^ (8) = 63.60, p = <.001. The post hoc comparisons revealed statistically significant differences in the relative frequencies of five of the nine categories (discussed in more detail below), whereas in the *Staffing*, *Physical environment and tools*, *Changing circumstances*, and *Own health and life situation* categories there were no differences in the relative frequencies between the two groups (Table 4). The responses under the five categories with statistically significant difference in relative frequencies were further examined to pinpoint the specific topics that were emphasised in each group.

**Table 4:** Observed frequencies, expected frequencies, and percentages within stress groups and total number and percentage of responses coded into each main category

Main category		High stress group	Low stress group	Total (%)
Cognitively strenuous conditions	Observed frequency	175^a^	101^b^	276 (5.2)
	Expected frequency	194	82	
	% within group	4.7	6.4	
Cooperation	Observed frequency	517^a^	326^b^	843 (15.8)
	Expected frequency	592	251	
	% within group	13.8	20.5	
Management and organisation of work	Observed frequency	989^a^	342^b^	1331 (25.0)
	Expected frequency	934	397	
	% within group	26.5	21.5	
Work overload	Observed frequency	736^a^	260^b^	996 (18.7)
	Expected frequency	699	297	
	% within group	19.7	16.4	
Staffing	Observed frequency	652^a^	286^a^	938 (17.6)
	Expected frequency	658	280	
	% within group	17.4	18.0	
Physical environment and tools	Observed frequency	158^a^	73^a^	231 (4.3)
	Expected frequency	162	69	
	% within group	4.2	4.6	
Changing circumstances	Observed frequency	235^a^	114^a^	349 (6.6)
	Expected frequency	245	104	
	% within group	6.3	7.2	
Clients	Observed frequency	194^a^	57^b^	251 (4.7)
	Expected frequency	176	75	
	% within group	5.2	3.6	
Own health and life situation	Observed frequency	81^a^	29^a^	110 (2.1)
	Expected frequency	77	33	
	% within group	2.2	1.8	
Total		3737	1588	5325 (100)

Different superscript letters (a, b) denote that the column proportions of the category differ significantly at a level of *p* < .05

#### Topics emphasised in the responses of the high stress group

The responses categorised under *Work overload*, *Management and organisation of work,* and *Clients* stood out in the responses of the high stress group. That is, their frequencies were higher than expected in the high stress group and lower than expected in the low stress group. Regarding the *Work overload* category, the topics emphasised in the responses of the high stress group mainly revolved around the experience of an excessive workload: too much work, too many clients, not having enough time for work tasks, working overtime, double shifts, and not having time to take statutory rest breaks. Typical responses were:

“Absurd amount of work. I estimate that I’m currently expected to do double the amount that was expected a year ago. (Reasons: change in process and team members' long absences.)”

“In the case of a sudden absence, instead of getting a substitute, the work is shared among the remaining employees.”

“Long workdays – after a morning shift you do the evening shift.”

“Lots of dealing with clients' errands that are not considered as belonging to working hours. [Medical record] entries and all these errands usually mean working overtime.”

Notions of ethical strain included in the *Work overload* category were also more common in the high stress group. These included comments such as:

“Nurses can’t work according to their own ethical standards.”

“Lack of time to do work tasks in accordance with one's own work ethic. You just have to carry on, even if you cut corners a bit. Most of all, these situations cause emotional strain.”

Interestingly, explicit talk about time pressure was equally prevalent in the responses of both stress groups.

Regarding the *Management and organisation of work* category, the respondents in the high stress group emphasised topics such as supervisors’ high demands and expectations, excessively wide-ranging duties and responsibilities, overly tight daily work schedules, and constant monitoring of work performance. Typical comments were:

“The inability of supervisors to put themselves into their employees' shoes. They know nothing about everyday work life. Requirements are constantly increased.”

“Reducing the number of employees even though the number of tasks increases.”

A striking aspect that was highlighted by the highly stressed employees was the unrealistic planning of daily work schedules. The employees commented:

“Unrealistic expectations: for example, three hours of work scheduled for between 8 am and 10 am, [and] not even travel time [from one client to another] is taken into account.”

“Rushing from one place to another. Not enough time is given for travel (by car) and driving conditions are not considered. For example, the customer might live 10 km away and the time reserved to get there is 5 minutes.”

Highly stressed employees also complained more about poor and unskilled supervisors in general and supervisors’ inappropriate behaviour or attitudes such as yelling, unfair treatment of employees, and even bullying. One employee commented that:

“Supervisor constantly disturbs, and bothers work. [Supervisor] singles me out and shouts when other employees are not present.”

With regard to the *Clients* category the respondents in the high stress group talked more about the poor health of clients and how it contributed to the heavy workload.

#### Topics emphasised in the responses of the low stress group

In the low stress group, responses categorised under *Cooperation* and *Cognitively strenuous conditions* stood out. That is, their frequencies were higher than expected in the low stress group and lower than expected in the high stress group. Regarding the *Cooperation* category, the respondents in the low stress group highlighted the uneven distribution of work tasks and responsibilities due to co-workers’ lack of contribution or skills, and the indifference of some employees with respect to workplace norms and practices. Typical comments were:

“Not everyone is able to cope with their tasks and this puts a strain on others.”

“Not all employees adhere to the agreed rules, and some are careless in their work, making the work a burden to others. Responsibility for work falls to only some members of the work team.”

“Not complying with ground rules in mutually agreed matters.”

Problems in the flow of information, a subcategory of *Cooperation*, were also emphasised somewhat more in the low stress group:

“New things keep coming up and information doesn’t reach everybody.”

“Conflicting or incomplete/missing information on clients' health or new instructions, etc.”

Regarding *Cognitively strenuous conditions,* the topics discussed were constant interruptions and distractions in daily work, such as:

“Noise in the workspace when trying to do paperwork.”

“Constant interruptions to work because the phone rings [or] when dealing with one client, three other clients interrupt with their own issues or demands. When working on [medical record] entries, answering the phone as well as other people giving a report in the same room interrupts work and breaks the flow.”

“Orientation of new employees (work is interrupted several times a day due to various questions).”

### Control over stressors

Finally, we interpreted and further categorised the main categories identified in the content analysis from the viewpoint of stressor controllability, that is, we identified the organisational actors who had control over the specific issues and situations mentioned in the responses. Thus, for each main category, we construed the level of the organisation on which specific hindrance stressors could be influenced. We ended up with four levels of control and assigned the nine main categories as follows:

1. Cooperating individuals and work teams: *Cognitively strenuous conditions, Cooperation*
2. Supervisors and managers: *Management and organisation of work, Work overload*
3. Directors and policymakers: *Staffing, Physical environment and tools*
4. Mainly circumstantial factors beyond the control of organisational actors: *Changing circumstances, Clients, Own health and life situation*

The interpretations were based on the content of the responses, as well as on a general understanding of the Finnish health care system. To give an example, the responses in the Work overload category included comments such as “supervisor’s unrealistic expectations of the amount of work” and “I’m expected to carry out double the amount of work I had a year ago”. Thus, the interpretations were partly readable straight from the responses and were strongly grounded in the data. However, not all the responses elaborated on the source of the demands, and some respondents merely commented, for example, that “there are too many clients per employee”. Therefore, we had to partly base the interpretations on a general understanding of how work is typically organised in the Finnish health care system. We know, for example, that the client/employee ratio is something over which individual employees usually have no control.

However, we also recognise that control over workplace stressors is seldom a black and white issue of full or no control, and therefore the grouping was not intended to be highly detailed but to roughly reveal who should be called upon to resolve the issues. The roles of supervisors, managers, and directors in particular are seldom as clear-cut as proposed above. For example, an individual supervisor’s or manager’s ability to influence work overload may be severely restricted and the issues may have to be solved on the level of directors and policymakers. Similarly, even though the main category of *Changing circumstances* is proposed to include mainly circumstantial factors that are beyond the reach of organisational actors, some of the issues discussed, such as frequent organisational changes, are more or less under the control of directors and policymakers.

According to the above categorisation, more than 70% of the overall content of the responses (see the final column of Table 4) dealt with stressors that were not in the hands of individual employees or work teams but mostly under the control of supervisors, managers, directors, and policymakers. These included issues such as excessive amounts of work, planning and scheduling of work and the related time pressure, and insufficient opportunities for recovery. Here, an important finding was that the excessive workload discussed by the employees was almost entirely framed as organisationally imposed (as opposed to self-imposed overload).

Issues that were directly related to and under the control of supervisors and managers were a major topic in 25% of the response content. These included problems relating to, for example, unclear instructions, ambiguously defined roles, uneven distribution of responsibilities, unequal or emotionally abusive treatment of employees, and lack of support. Hindrance stressors that may be influenced by cooperating employees and work teams were reflected in about 20% of the overall content of the responses, and these included poor flow of information, unnecessary interruptions and distractions, and lack of commitment to common work practices. Although supervisors, managers, and organisational practices can support cooperation and facilitate improvements in cognitive working conditions, these factors rely on individual-level and team-level behaviour.

Finally, there were differences between the groups of high and low stress in terms of the actors who typically have control over the issues highlighted in the responses of each group. Respondents in the high stress group emphasised stressors that were mainly under the control of supervisors and managers and thus reflected working conditions over which individual employees or work teams usually had little control. In contrast, the responses of the low stress group more frequently reflected factors that concerned or were under the control of individual employees and work teams.

## Discussion

The results of our study provide an overall picture of the numerous factors that employees working in the health care sector perceive as stressors that hinder their flow of work. The results also showed that employees with high and low levels of subjective stress emphasised partly different topics. Moreover, our interpretation and categorisation of the organisational actors who have control over the stressors suggests that the most significant hindrance stressors were to a large extent beyond the control of individual employees and work teams.

### Automated content analysis captures the voices of employees

The unique value of our study resides in its use of semi-automated, data-driven analysis, which enabled us to draw conclusions regarding how the major hindrance stressors clustered and overlapped in the extensive textual data produced by the employees. The results of the semi-automated analysis were contrasted with the results of the conventional content analysis in which a subset of the same textual data was coded by the first author. The main results of the two methods of analysis were largely overlapping, but they also provided unique perspectives on the data.

Concerning our first research question, the results highlighted a variety of hindrance stressors that compromise the flow of work. The most significant stressors were inadequate staffing levels, work overload, and constant time pressure, all of which previous studies have also recognised as major stressors (Bennett, 2001, Bowling & Kirkendall, 2012; Hannigan et al., 2004; Mazzola et al., 2011; McVicar, 2003; Smollan, 2015). Another prominent and interlinked cluster of themes present in the responses were notions of constantly changing situations, unclear instructions, interruptions, and noisy work environments, which have also been identified as stressors in previous studies (e.g., Elfering et al., 2011). Finally, a central theme of management was highly interconnected with many of the other topics, which is in line with previous studies that have found that poor supervisor–employee relations are related to higher strain among employees (e.g., Gilbreath & Benson, 2004; Tepper, 2000).

Our results revealed multiple significant hindrance stressors that have seldom been covered in a single study. Our results thus added to previous results by showing that many themes co-occurred within the responses, suggesting that these issues intertwine in everyday work and form a complex network of interconnected stressors. Many responses also explicitly reported causal relationships among the major hindrances. For example, inadequate staffing levels emerged as a root cause of many of the other issues. This kind of complexity of psychosocial factors that underlie harmful stress is challenging for quantitative research that models specific theoretical constructs. Our results indicate the importance of modelling a larger set of psychosocial risk dimensions than what any current theory of work stress approaches. The conceptual and methodological limitations in psychosocial occupational research and the need to expand research on the psychosocial working environment beyond current theoretical frameworks have also been discussed by Burr et al. (2016). Acknowledging the many intertwining hindrance stressors may also be essential from the perspective of developing stress management strategies that have a broader focus that can effectively handle the complex of stressors.

Regarding the second research question, the key findings revealed differences between the responses of employees who reported high stress levels and of those who reported low stress levels. A difference, in terms of quantity, was that the respondents in the high stress group raised relatively more issues in their responses than those in the low stress group. This is in line with recent findings showing that co-occurring work stressors, rather than any single stressor, are harmful and may risk work ability (Juvani et al., 2018). In terms of content, employees in the high stress group emphasised concerns about excessive amounts of work, high expectations and demands of supervisors, tightly scheduled workdays, role overload, and clients who needed more time and care than they were able to provide. Many striking examples in the responses highlighted the impossible situations that the employees were faced with in their daily work when trying to fit time-consuming work tasks into their schedules which, in reality, did not provide the time required to perform the tasks.

Our results thus provide detailed examples of the many concrete demands and situations that underlie the experience of stress at work. Our findings demonstrate the importance of filling the gap in the literature raised by Lukan et al. (39). They argue that, exploring day-to-day stressors in more detail, rather than studying broad constructs such as decision latitude, helps us understand how particular stressors lead to health outcomes, and facilitates the development of stress management strategies that are applicable in everyday work life.

### Most hindrance stressors are beyond the control of employees and work teams

With regard to the third research question, our findings uncovered the actors at different organisational levels who can typically influence the major hindrance stressors. A finding of particular concern was that the work overload and related time pressure, frequently discussed especially in the responses of the high stress group, were almost entirely organisationally imposed. Thus, the employees had no control over the overload, which has been shown to be especially exhausting in comparison to self-imposed work overload (Laurence et al., 2016). Overall, our results indicate that the majority of the hindrance stressors mentioned in the responses cannot be influenced by individual employees, work teams, or necessarily even supervisors, and that they require actions of people from higher levels of the organisation. Several stressors were mentioned that require actions from those who make strategic decisions in the organisations or even from policymakers. Indeed, many of the concerns reflected larger societal issues and structural problems, such as the allocation of resources to the health care sector.

Overall, the topics emphasised by the high stress group highlighted the role of supervisors in designing realistic job demands, when the aim is to reduce harmful stress. The responses of the group that reported low levels of stress, on the other hand, offered valuable insights into how cooperating employees and work teams, with the support of supervisors and managers, can proactively influence working conditions. The results call for workplace development and interventions that target poor flow of information, unclear or ambiguous instructions, unnecessary interruptions and distractions, and a lack of common work practices or commitment to existing ones. Improving these issues could immediately alleviate employees’ stress. These kinds of stressors can be tackled by regularly discussing the issues and committing to practices that facilitate everyday work. Our results thus suggest that focusing on ergonomics and designing better working conditions may be a useful stress management strategy that is applicable in everyday work life and even more effective than individual level stress management approaches (Kalakoski et al., 2020).

### Limitations and suggestions for future research

Despite the contributions of the current study, it is not without limitations. Although our sample was extensive and diverse, and included respondents from a variety of speciality fields and occupations, we only focused on work in the health care sector. Furthermore, the results were based on open-ended questionnaire responses, and certain specialisation fields and groups of workers were over-represented, all of which limit the generalisability of our results within and outside the health care sector. We recommend that future studies use randomly selected samples and complementary methods, such as repeated and continuous assessment, carried out in day-to-day work and environments (see also Lukan et al., 2022). Moreover, our data were collected before the COVID-19 pandemic, and future studies are needed to show whether new (post) pandemic hindrance stressors have emerged.

The cross-sectional nature of the data also prevents us from drawing conclusions about causal relationships between the described working conditions and self-reported stress. Therefore, longitudinal studies of the topic are also needed. There is also a need for multidimensional stress scales that provide more detailed information on how specific working conditions are related to different manifestations and sub-dimensions of psychosocial stress. However, the single item stress measure used in our study has shown satisfactory content, criterion and construct validity and was thus a valid replacement for longer scales (Elo et al., 2003). Nevertheless, using only self-report assessments is subject to common-method variance and subjectivity bias; for example, some respondents may have exaggerated problems both in their stress levels and in their work environment, which can lead to spurious findings (Theorell & Hasselhorn, 2005). However, the data used in our study were extensive, and the respondents came from many different organisations and occupational settings, and therefore, the issues that they repeatedly raised are highly likely to reflect actual problems in working conditions.

Moreover, even if there is strong evidence that self-reported stress is a risk factor for a multitude of adverse health outcomes such as physiological disease outcomes and mental health problems (e.g., Kivimäki et al., 2002; Tennant, 2001), future studies should combine the descriptions of workplace stressors with objective (physiological) measures of stress or continuous measurements that are not subject to memory bias (Lukan et al., 2022) and that reveal stress-related processes that operate beyond our awareness (Brosschot, 2010).

Finally, there were some indications that the actor–observer effect (i.e., the tendency to attribute one’s own behaviour to external causes and that of others to internal causes), may have affected our data, even if recent research has suggested that the effect is small or even non-existent (Malle, 2006). Indications of this effect were evident, for example, in how especially employees in the low stress group, discussed actions and non-actions of co-workers, which they mainly framed as being connected to internal causes such as indifference to mutually agreed-on practices or even laziness, as a burden to themselves. Therefore, there is a need to explore whether, for example, supervisors have similar perceptions to those of employees with respect to the issues that most centrally hinder the flow of work.

As the results of the semi-automated and conventional content analysis largely converged with each other and with earlier findings, the results demonstrate that modern text mining methods enable the discovery of central topics in much larger textual data sets than those usually used in qualitative research. Automated analyses of open-ended responses and other textual data open up new opportunities to utilise substantial amounts of material gathered not only in research settings but also in workplace wellbeing surveys or piling up on business communication platforms. Future studies could use large textual sources of information to identify new kinds of stressors when they are only just beginning to appear in the responses and discussions of employees. In this way, it could be possible to anticipate the need for better solutions and interventions already before the harmful consequences of stress manifest.

Our results concerning control over hindrance stressors suggest that future studies should pay more attention to the organisational actors who have control over the conditions that cause impaired workflow. The controllability of common stressors in different occupational settings should also be explored and individual appraisals of controllability incorporated into explanatory models. For example, it would be useful to develop and validate a scale of stressor controllability, as in the current methods that include positive, negative, and threat appraisals attached to specific stressors (e.g., Smith et al., 2020). Work stress theories should also consider the extent to which the psychological resources that are often provided as the first (and sometimes even the only) solution can buffer against the hindrance stressors that seem to be largely beyond employee control. Is it possible that attempts to increase an individual’s psychological resources may, under certain conditions, even increase their level of stress, by pointing the finger at an employee who is already performing ‘mission impossible’?

### Theoretical and practical implications

The study makes important theoretical contributions to the literature on work stress. With regard to the CHM, our results lend further support to the critique of the use of a priori classifications (e.g., Bakker & Sanz-Vergel, 2013; Horan et al., 2020) as many stressors traditionally framed as motivating challenges in the CHM framework were clearly hindrance stressors for our extensive group of respondents, regardless of whether the respondent’s general level of stress was high or low. Hence, our findings further emphasise the importance of taking individual appraisal into account whenever making conclusions about stressors being challenges or hindrances which may have differing effects on well-being.

Moreover, the results of the current study, like those of a vast amount of modern (neuro) physiological stress research (e.g., Koolhaas et al., 2011; Limbachia et al., 2021; Meine et al., 2021), suggest that stressor controllability is a significant factor that should be more explicitly incorporated in current psychological models of work stress. For example, JD-R currently recognises on a general level that job control and/or autonomy are important job resources (Bakker & Demerouti, 4), and research distinguishes between different dimensions of autonomy (De Spiegelaere et al., 2016). However, the experience of control over specific stressors has not been incorporated in the CHM as a mechanism that can mediate the stressor-strain relationship. We argue that future models should consider the inclusion of control appraisal as something that is explicitly linked to specific everyday job demands. For example, the respondents could be asked separately about any given stressor to what extent they perceive to have control over it, and not only whether it is a challenge, a hindrance, or a threat (e.g., Tuckey et al., 2015). The focus of theoretical models of psychosocial risk factors needs to be expanded to day-to-day working conditions (Lukan et al., 2022) and a larger variety of stressors (Burr et al., 2016), but also to integrate the appraisal of stressor controllability.

Our study also has several practical implications with regard to stress management practices. Based on the findings we suggest that in the efforts to improve the flow of work and to manage work-related stress, supervisors, managers and even directors need to be more powerfully engaged since they are the ones who have control over the common sources of impaired workflow and strain in daily work. The factors emphasised in the responses also cry out for changes in resources and structures which directors and policymakers have to make. In other words, we need supervisors, managers, directors, and policymakers to step in and handle the stressors and improve working conditions that are beyond the control of employees. In contrast, commonly used individual-level interventions that aim to improve employees’ stress management skills and health behaviours do not seem sufficient from the viewpoint of the problems highlighted in our results, such as the overwhelming workloads and pressures that employees face.

We need stress prevention that focuses on reducing hindrance stressors and on creating working conditions that support both employee wellbeing and the flow of work. Focusing on stressors instead of the appraisals and coping strategies of employees is an often ignored but potentially highly effective approach to stress prevention (e.g., Rickard et al., 2012). Indeed, more comprehensive stress management frameworks that approach stress with an emphasis on not only the individual employee but also on improving multiple parts of the larger organisational system have been proposed in recent years, and these often highlight the involvement of employees in a way that increases their autonomy and control over job demands (e.g., Grawitch et al., 2015). If the detrimental effects of work stress are to be reduced, we need interventions that acknowledge the various sources of stress at work and engage employees from all organisational levels, and even policymakers, when needed.

## Conclusion

Our study revealed a host of hindrance stressors that impair the daily flow of work in the health care sector. The majority of stressors emphasised by employees with high levels of subjective stress were beyond their control and were to a great extent controlled by supervisors and managers. Many of the common stressors in the psychosocial work environment also reflected extensive structural problems, such as employee shortages and work overload, which the health care sector is facing in general. However, issues that can be tackled by committing to certain actions and work practices on individual and team levels, with the support of supervisors, were also brought up in the responses. Our results suggest that controllability is a significant feature of stressors that should be studied in more detail in the context of current work stress models. Our findings also indicate that seeking to diminish the effects of work-related stress on only the individual or team level is not enough; increasing the psychological resources of individual employees should not be overemphasised in stress management. Interventions are needed that target the actual stressors and thus aim to improve the working conditions and poor work practices that cause stress. The support of supervisors and the decisions and actions of directors and policymakers are also of critical importance if the detrimental effects of stress on individuals, organisations, and society are to be reduced.

## Data Availability

The data sets analysed during the current study and more detailed descriptions of the applied analysis procedures are available from the corresponding author on reasonable request.

## Appendix

The Leximancer analysis was conducted exploratively, using unsupervised analysis. The software was set to identify only word-like concepts, and the number of sentences processed together (i.e., text segment size) was set to two sentences, which is a default setting. Despite the fixed two-sentence length of text segments, Leximancer does not mix responses from different respondents, that is, for responses containing only one sentence, the text segment size was naturally smaller. Furthermore, the text segments were prevented from crossing paragraph boundaries. To take into account the many filler words and words otherwise meaningless in the context of the study, such as “quite” and “yet”, used in the responses, the authors used Leximancer’s default Finnish stop word list and complemented it with some additional words.

In the first stage of the analysis, the Leximancer default algorithm was used to identify the naturally emerging number of concepts from the text. A total of 75 concepts were identified and their frequency of occurrence in the text ranged between 43 and 1001. The fine-tuning of the concept list, a necessary procedure in unsupervised content analysis with Leximancer (Crofts & Bisman, 16), was conducted by carefully assessing the meaning of the identified concepts combined with the occurrence and co-occurrence information provided by the software. In the fine-tuning process, we merged the inflected forms of words typical to Finnish language, synonyms, plurals, and words with similar meanings (e.g., manager and managers; sufficient and enough; staff and employees). We also removed concepts with low semantic meaning (e.g., during, often, really) or that were too general (e.g., own, things) from analysis. After the concept list was fine-tuned, the number of remaining concepts was 31, with counts ranging between 68 and 1153.

In the second stage, these 31 concepts were compared against a list of 200 most frequent words in the responses (generated with ATLAS.ti). This list included several words that were potentially relevant in our context but did not appear in the initial Leximancer analysis (e.g., information flow, workplace atmosphere, absences). Therefore, we ran through the Leximancer analysis again and set the number of concepts to be extracted to 200. After a similar fine-tuning process to that described above (i.e., merging and removing concepts), the concepts with counts considerably lower than that of the least prevalent concept in the initial automatic analysis were removed as potential “junk concepts”. These represent concepts that may emerge in the analysis when Leximancer is forced to look for more concepts than is naturally emergent in the data. The resulting final number of concepts was 55, with counts ranging between 40 and 1556. Theme size was set to 40%, and this yielded nine distinct themes into which the concepts were clustered.
